# Left Variant Atypical Atrioventricular Nodal Reentrant Tachycardia Associated With Situs Inversus Totalis

**DOI:** 10.1016/j.jaccas.2026.108595

**Published:** 2026-05-29

**Authors:** Yuki Tanaka, Itsuro Morishima, Yasunori Kanzaki, Kazuki Shimojo

**Affiliations:** Department of Cardiology, Ogaki Municipal Hospital, Ogaki, Japan

**Keywords:** atrioventricular nodal reentrant tachycardia, catheter ablation, electroanatomical mapping, left variant slow pathway, situs inversus totalis

## Abstract

**Background:**

Left variant type slow–slow atrioventricular nodal reentrant tachycardia (AVNRT) is rare, and its occurrence in patients with situs inversus totalis has not been previously reported.

**Case Summary:**

A patient with situs inversus totalis presented with recurrent supraventricular tachycardia. Electrophysiological study excluded atrioventricular reentrant tachycardia and atrial tachycardia, supporting a diagnosis of left variant slow–slow AVNRT. Catheter ablation targeting the left atrial slow pathway at the inferolateral mitral annulus was performed using cardiac computed tomography and high-density electroanatomical mapping. Tachycardia was terminated with a single radiofrequency application and was no longer inducible.

**Discussion:**

Catheter ablation in patients with situs inversus is technically challenging because of mirror-image anatomy, but advanced imaging and electroanatomical mapping allow accurate localization of the arrhythmogenic substrate and safe ablation.

**Take-Home Messages:**

Left variant slow–slow AVNRT can occur in patients with situs inversus totalis. Advanced imaging and electroanatomical mapping facilitate safe ablation despite anatomical complexity.

## Visual Summary

**Figure:**
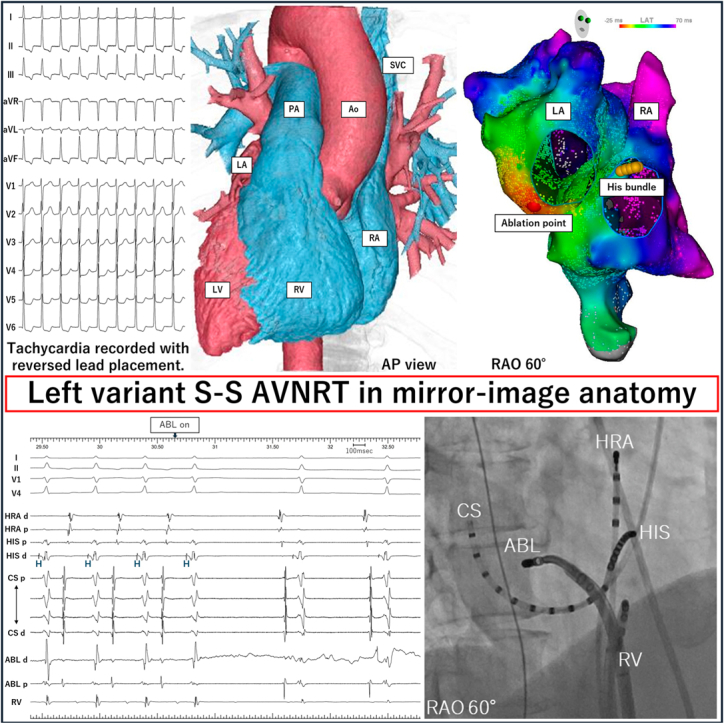

Situs inversus totalis is a rare congenital condition, occurring in approximately 1 in 10,000 individuals.^1^ In patients with situs inversus, the coexistence of supraventricular tachycardia is uncommon and may present additional technical and diagnostic challenges during electrophysiological evaluation and catheter ablation. The left variant form of atrioventricular nodal reentrant tachycardia (AVNRT) itself is rare, with a reported prevalence of approximately 1.2%.^2^ To the best of our knowledge, no prior reports have described left variant type slow–slow AVNRT in association with situs inversus totalis. This combination represents an extremely rare and technically challenging substrate.

## History of Presentation

**Figure 1:**
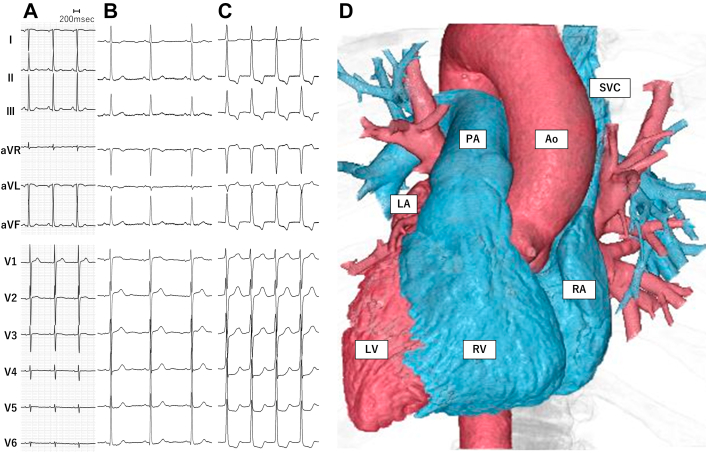
12-Lead Electrocardiograms and 3-Dimensional Cardiac Computed Tomography (A) Twelve-lead electrocardiogram recorded during sinus rhythm with the standard lead configuration. (B) Sinus rhythm recorded with reversed lead placement. (C) Tachycardia recorded with reversed lead placement. (D) Three-dimensional cardiac computed tomography in the anteroposterior view demonstrating situs inversus totalis. Ao = aorta; LA = left atrium; LV = left ventricle; PA = pulmonary artery; RA = right atrium; RV = right ventricle; SVC = superior vena cava.

A 59-year-old woman with a congenital diagnosis of situs inversus totalis was referred to our hospital for catheter ablation of drug-refractory narrow QRS complex tachycardia. Despite treatment with verapamil, her symptoms remained inadequately controlled. She had no history of cardiac disease or prior cardiac surgery, and there was no family history of sudden cardiac death or inherited arrhythmia syndromes. Physical examination revealed no signs of heart failure. Electrocardiography during sinus rhythm and 3-dimensional cardiac computed tomography (CT) demonstrated findings consistent with situs inversus totalis (Figure 1).

## Investigations

An electrophysiological study was performed. Baseline intracardiac recordings demonstrated sinus rhythm with an AA interval of 708 ms, AH interval of 98 ms, and HV interval of 48 ms. Dual atrioventricular nodal physiology was observed during antegrade conduction. Para-Hisian pacing from the right ventricle resulted in prolongation of the Stim–A interval with loss of His capture, whereas the HA interval and atrial activation sequence remained unchanged, indicating an atrioventricular nodal/atrioventricular nodal response. The earliest atrial activation during retrograde conduction was recorded at the posterolateral coronary sinus (CS) and exhibited decremental conduction properties. The clinical tachycardia was induced by constant pacing from the right ventricle with a pacing cycle length of 400 ms. The tachycardia cycle length was 444 ms, with an AH interval of 218 ms, HA interval of 226 ms, and VA interval of 152 ms. The atrial activation sequence remained consistent with that seen during retrograde conduction.

**Figure 2:**
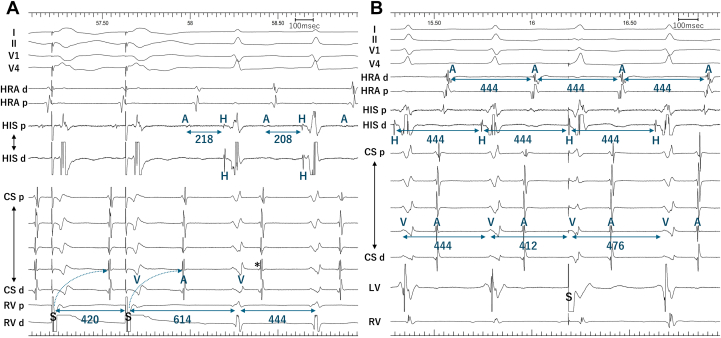
Intracardiac Electrograms During Tachycardia (A) Ventricular overdrive pacing performed at a pacing cycle length of 420 ms during tachycardia with a cycle length of 444 ms. Pacing impulses propagated retrogradely through the slow pathway and captured the atrium (dotted arrows). The tachycardia response demonstrated a V–A–V pattern. The postpacing interval minus tachycardia cycle length (PPI–TCL) was 170 ms, and the corrected PPI–TCL was 160 ms. During tachycardia, the earliest atrial activation electrogram consisted of an initial dull component followed by a sharp component (∗). Surface electrograms were recorded with reversed lead placement. (B) His-refractory premature ventricular contractions delivered from the left ventricle. The ablation catheter was positioned trans-septally in the left ventricle, and pacing was performed. Ventricular timing adjacent to the earliest atrial activation was advanced, without resetting either the HH interval or the AA interval. Surface electrograms were recorded with reversed lead placement. CS = coronary sinus; d = distal; HIS = His bundle; HRA = high right atrium; p = proximal; S = stimulus; Other abbreviations as in Figure 1.

His-refractory premature ventricular contractions failed to reset the AA or HH intervals. Right ventricular overdrive pacing at a cycle length of 420 ms produced a V–A–V response with a postpacing interval minus tachycardia cycle length of 170 ms and a corrected postpacing interval minus tachycardia cycle length of 160 ms (Figure 2A). These findings excluded orthodromic reciprocating tachycardia and atrial tachycardia, suggesting AVNRT. To further exclude orthodromic reciprocating tachycardia via a left-sided slowly conducting accessory pathway, His-refractory premature ventricular contractions were delivered from the left ventricle using a trans-septally positioned ablation catheter. The absence of tachycardia resetting argued against the presence of a left-sided accessory pathway (Figure 2B). Given the prolonged AH (>200 ms) and HA (>70 ms) intervals, the tachycardia was diagnosed as left variant type slow–slow AVNRT.

## Management

**Figure 3:**
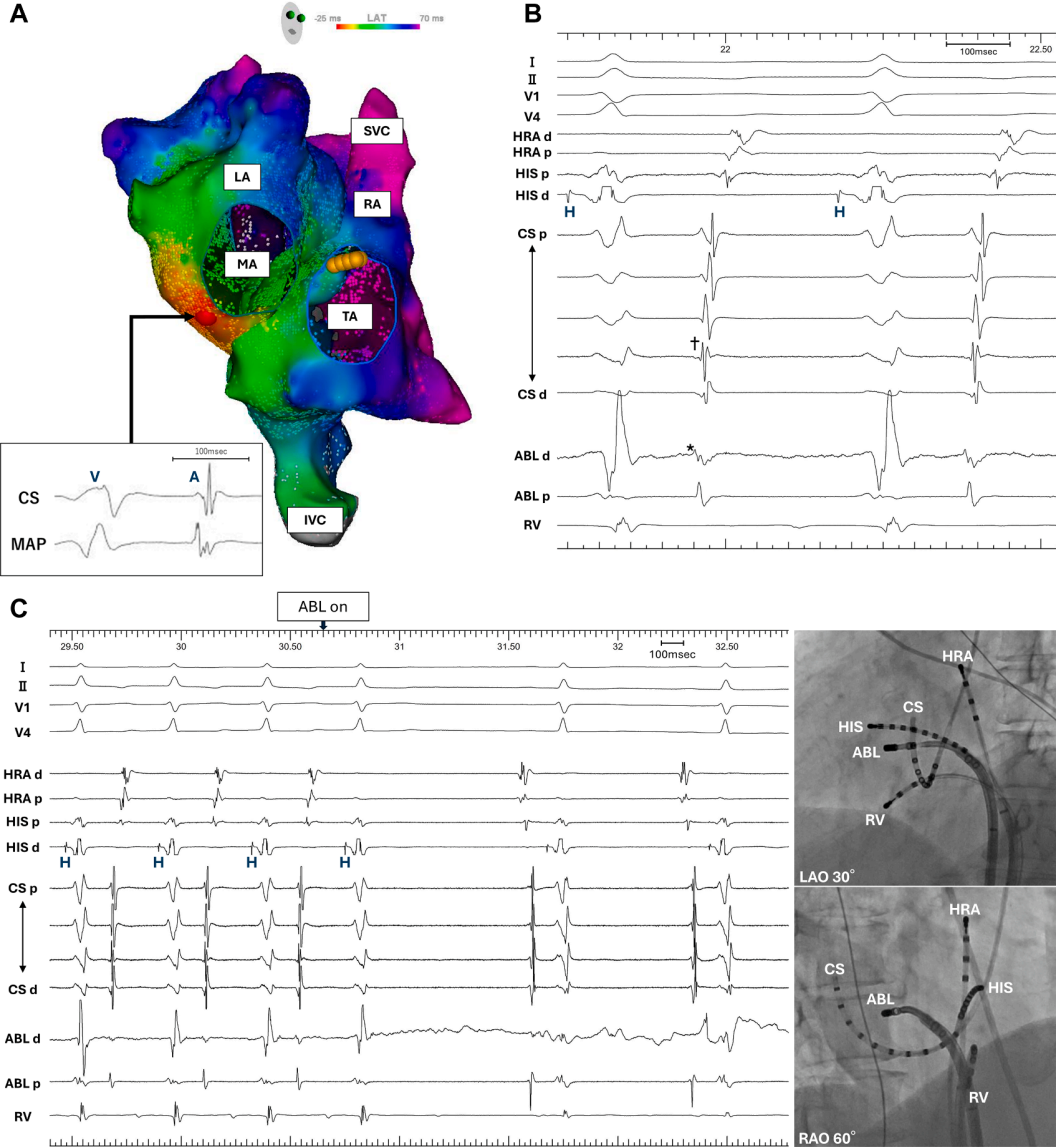
Three-Dimensional Electroanatomical Mapping and Ablation (A) Biatrial propagation map during tachycardia. The yellow tag indicates the His bundle, and the red tag marks the earliest atrial activation site at the 8 o’clock position of the mitral annulus. At this site, a sharp–dull atrial electrogram was recorded on the left atrial aspect by the mapping catheter. At the opposing coronary sinus site, a dull–sharp electrogram pattern was observed, indicating later activation on the coronary sinus side. (B) Intracardiac electrogram at the earliest atrial activation site during tachycardia. The ablation catheter was positioned at this site via a trans-septal approach, where the local atrial electrogram (∗) preceded the coronary sinus potential (†) by 12 ms. Surface electrograms were recorded with reversed lead placement. (C) Intracardiac electrogram during radiofrequency ablation at the earliest atrial activation site. Energy delivery during tachycardia resulted in termination of the tachycardia within 0.3 s with ventriculoatrial block. Surface electrograms were recorded with reversed lead placement. ABL = ablation catheter; IVC = inferior vena cava; LAO = left anterior oblique; MA = mitral annulus; MAP = mapping catheter; RAO = right anterior oblique; TA = tricuspid annulus; Other abbreviations as in Figure 1, Figure 2.

Because the earliest atrial electrogram recorded at the posterolateral CS consisted of an initial dull component followed by a sharp component (Figure 2A), high-density 3-dimensional electroanatomical mapping of both atria was performed using a multispline mapping catheter (Octaray; Biosense Webster). The earliest atrial activation was identified at the 8 o’clock position of the mitral annulus, opposite the earliest CS activation site, and preceded CS activation by 12 ms (Figures 3A and 3B). Targeted radiofrequency ablation at this site resulted in immediate termination of the tachycardia and elimination of ventriculoatrial conduction (Figure 3C).

## Outcome and Follow-Up

A postablation electrophysiological study under isoproterenol infusion demonstrated restored retrograde nodal conduction via the fast pathway, without evidence of retrograde slow pathway conduction. Tachycardia was no longer inducible, and the procedure was concluded. During follow-up, the patient remained free from tachycardia recurrence without antiarrhythmic therapy.

## Discussion

The final diagnosis was left variant type slow–slow AVNRT, supported by the following findings: prolonged AH (≥200 ms) and HA (≥70 ms) intervals during tachycardia, earliest atrial activation at the posterolateral mitral annulus, a V–A–V response during ventricular overdrive pacing, and lack of tachycardia resetting with His-refractory premature ventricular contractions. Otomo et al^3^ reported that in atypical AVNRT with eccentric CS activation, the dull component of the atrial electrogram recorded within the CS represents far-field potentials from the left atrium (LA), whereas the sharp component reflects near-field potentials from the CS musculature. They noted that the dull and sharp components recorded at the earliest CS activation sites reflect initial activation at the atrial insertion of the left-sided slow pathway, followed by subsequent activation of the CS musculature via the LA–CS connection.^3^ In our case as well, distinct dull and sharp components were observed at the earliest CS activation site during tachycardia. In addition, in the LA activation map, the earliest activation site preceded the CS by 12 ms, suggesting the atrial insertion of the slow pathway was located on the LA side (Figures 3A and 3B). Although Otomo et al^3^ reported that ablation from within the CS is sufficient for successful slow pathway elimination in approximately 75% of cases with a left variant slow pathway,^4^ the electrophysiological findings in the present case suggested that ablation from the LA side would be more appropriate. As a result, tachycardia was successfully terminated with a single radiofrequency application.

Catheter ablation in patients with situs inversus can be technically challenging because of mirror-image anatomy. Previous studies have proposed strategies such as mirror-image placement of 12-lead electrocardiographic electrodes and modification of fluoroscopic views, including horizontal image inversion and interchange of right and left anterior oblique projections.^5^^,^^6^ In this case, careful fluoroscopic orientation combined with cardiac CT integration and high-density electroanatomical mapping enabled safe, precise catheter manipulation and successful ablation.

## Conclusions

We report a rare case of left variant type slow–slow AVNRT associated with situs inversus totalis. Despite the anatomical complexity, successful and safe catheter ablation was achieved through the targeted integration of cardiac CT and 3-dimensional electroanatomical mapping.

**Visual Summary:**
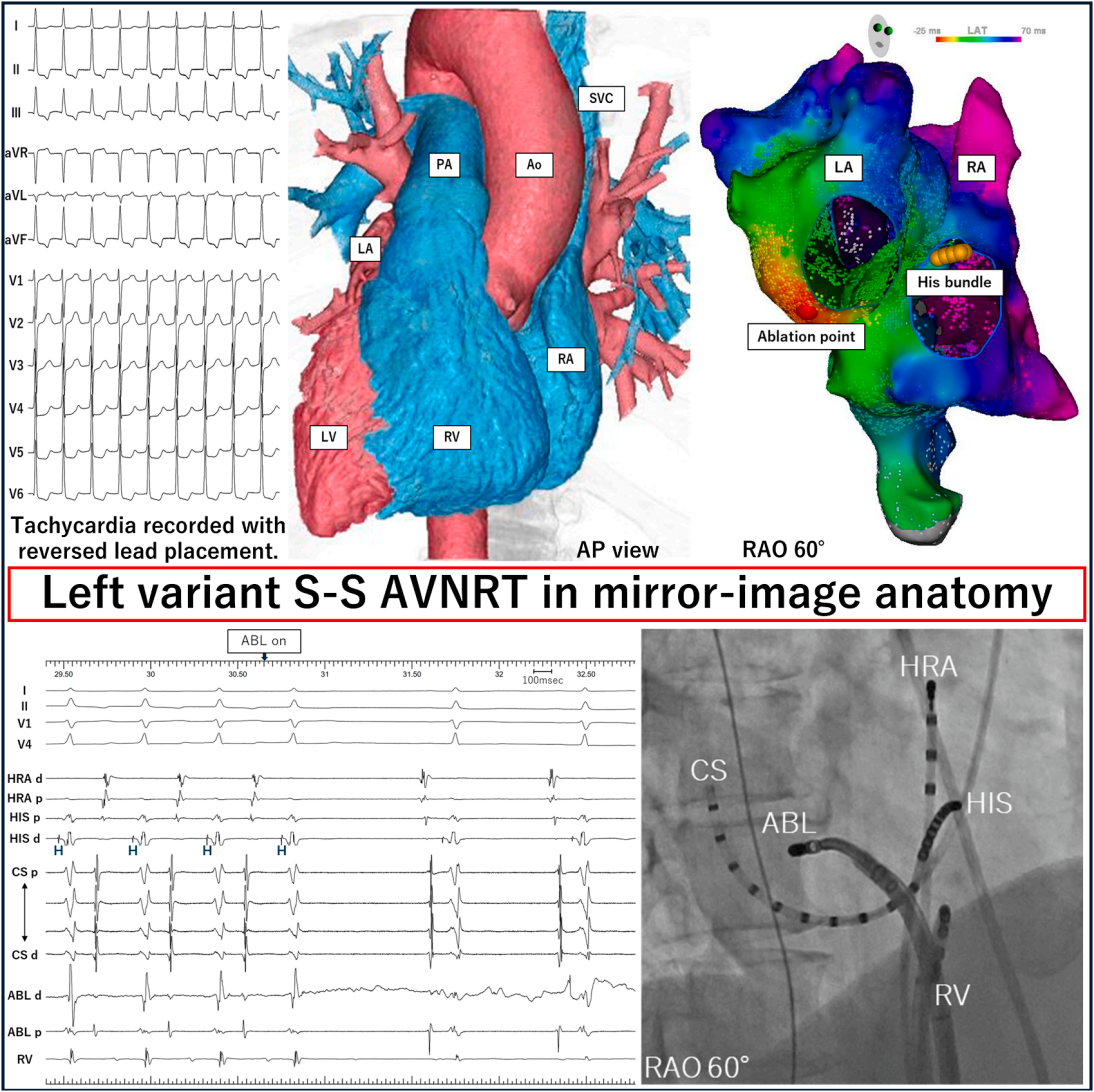
A Rare Case of Left Variant Type Slow–Slow Atrioventricular Nodal Reentrant Tachycardia in a Patient With Situs Inversus Totalis Is Presented Integration of cardiac computed tomography and high-density 3-dimensional electroanatomical mapping enabled accurate localization of the left atrial slow pathway at the inferolateral mitral annulus and successful catheter ablation despite mirror-image anatomical complexity. ABL = ablation catheter; Ao = aorta; A→P = anteroposterior; AVNRT = atrioventricular nodal reentrant tachycardia; CS = coronary sinus; HIS = His bundle; HRA = high right atrium; LA = left atrium; LV = left ventricle; PA = pulmonary artery; RA = right atrium; RAO = right anterior oblique; RV = right ventricle; S-S = slow–slow; SVC = superior vena cava.

## Funding Support and Author Disclosures

The authors have reported that they have no relationships relevant to the contents of this paper to disclose.

**Take-Home Messages**

• Left variant type slow–slow AVNRT, although rare, should be considered in patients with situs inversus totalis presenting with supraventricular tachycardia.
• Advanced imaging and 3-dimensional electroanatomical mapping facilitate safe and effective catheter ablation in the presence of complex mirror-image anatomy.
